# The akinetic crisis in Parkinson´s disease- the upper end of a spectrum of subacute akinetic states

**DOI:** 10.1007/s00702-024-02817-8

**Published:** 2024-08-17

**Authors:** Monika Pötter-Nerger, Christoph Schrader, Wolfgang H. Jost, Günter Höglinger

**Affiliations:** 1 Department of Neurology, University Medical Center Hamburg-Eppendorf, Martinistraße 52, 20246 Hamburg, Germany; 2 Department of Neurology, Medical School Hannover, Hannover, Germany; 3 Center for Movement Disorders, Parkinson-Klinik Ortenau, Wolfach, Germany; 4 Department of Neurology, LMU University Hospital, Ludwig-Maximilians-Universität (LMU) München, Munich, Germany; 5 German Center for Neurodegenerative Diseases (DZNE), Munich, Germany; 6 Munich Cluster for Systems Neurology (SyNergy), Munich, Germany

**Keywords:** Akinetic crisis, Spectrum acute akinetic states, Risk factors, Cytokines, Therapy, Parkinson’s disease, Neuroleptic malignant-like syndrome, Parkinsonism-hyperpyrexia syndrome

## Abstract

The akinetic crisis is defined as an acute, potentially life-threatening, levodopa-resistant, severe aggravation of rigidity, severe akinesia, associated with high fever, disturbance of consciousness, dysphagia and autonomic symptoms often due to disruption of dopaminergic medication or infections. The akinetic crisis is a relatively rare event, however subacute mild-moderate motor symptom deterioration in Parkinson´s disease (PD) patients is a frequent cause of hospitalization. In this review, we propose that the akinetic crisis is the upper end of a continuous spectrum of acute akinetic states depending on the degree of the progressive levodopa-resistance. Clinical symptomatology, risk factors, and instrumental diagnostics as the DAT-SPECT reflecting a biomarker of levodopa-resistance will be discussed to evaluate the spectrum of akinetic states. Pathophysiological considerations about the potential role of proinflammatory cytokines on the progressive levodopa-resistance will be discussed and therapeutical, consensus-based guidelines will be presented.

## Introduction

The akinetic crisis in its full clinical presentation is a rare event in about 0.3% of Parkinson´s disease (PD) patients/year (Onofrj et al. 2009; Onofrj and Thomas 2005). It is defined as an acute, potentially life-threatening worsening of symptoms in PD patients with transient resistance to dopaminergic medication. Narrower criteria for defining akinetic crisis have been proposed as subacute worsening of UPDRS > 20 points accompanied by transient resistance to dopaminergic medication for > 3 days (Thomas and Onofrj 2005). Acute akinetic crisis is also often referred as “Parkinson’s hyperpyrexia syndrome”, “malignant syndrome”, “acute akinesia” or “neuroleptic malignant-like syndrome”, based on historically observed similarities with psychiatric patients on high-dose neuroleptic medication who experience fever and severe akinesia in the sense of a “malignant neuroleptic syndrome“(Kipps et al. 2005).

The akinetic crisis in its complete presentation is a rare event, however in the clinical everyday routine, clinicians treat very often PD patients with acute worsening of clinical symptoms seeking emergency hospitalization(Zheng et al. 2012), but who do not fulfill all characteristics defining the akinetic crisis. Very often, PD patients with acute worsening of symptoms are admitted to hospital without any preceding changes of their medication, but with e.g. urinary tract infection and concomitant increase of symptom load(Brugger et al. 2015). In those patients with an “almost-akinetic crisis”, the clinical demand to augment therapeutically the dopaminergic dosage points to a common mechanism of a beginning dopamine resistance. We propose a spectrum of acute-subacute akinetic states depending on the degree of the dopamine resistance with the “full-picture” akinetic crisis as the upper, worst end of that spectrum.

### Clinical characteristics of the akinetic crisis

The clinical symptomatology of a “full-picture” akinetic crisis is associated with specific stigmata. Essentially, there is the fulminant aggravation of rigidity and severe akinesia with a mean worsening in the Hoehn & Yahr stage from 2 to 4–5 (Onofrj and Thomas 2005) leading to bedriddenness (Table 1). A second, most often observed symptom is high fever, so that some authors consider the presence of an elevated core body temperature to be the most important diagnostic criterion for an akinetic crisis (Takubo et al. 2003). Further symptoms include disturbance of consciousness in about half of the patients, a state of confusion, loss of appetite, dysphagia, a general feeling of illness and autonomic symptoms such as increased sweating, hypersalivation, and blood pressure fluctuations(Onofrj and Thomas 2005; Takubo et al. 2003). Other case reports describe increased tremor, dystonic symptoms (Wang et al. 2022), and rarely myoclonus(Takubo et al. 2003).

The clinical course is a subacute-acute worsening of motor symptoms within 2–3 days, duration of about 11 days and recovery after 4–26 days (Onofrj and Thomas 2005), but there are also descriptions of associated mortality of 4–23% (Onofrj and Thomas 2005; Takubo et al. 2003; Wang et al. 2022; Bonanni et al. 2014). There are several, life-threatening complications of the akinetic crisis, that contribute to the high mortality risk as rhabdomyolysis (Jayaram and Chancellor 1997), aspiration pneumonia (19.2%), disseminated intravascular coagulation disorders (8.1%) and acute renal failure (5.1%)(Takubo et al. 2003). Further complications may include thrombocytopenia, respiratory failure (Sahu et al. 2018), very rarely epileptic seizures, acute liver failure, acute cardiac decompensation (< 2%) (Takubo et al. 2003) or cardiac arrhythmias. Furthermore, a “critical illness neuromyopathy” with flaccid tetraparesis and a renewed increase in muscle enzymes has been described as a complication of an akinetic crisis (Capasso et al. 2015).

This full clinical, dramatic picture of the akinetic crisis is rare, however a subacute worsening of akinesia potentially with fever, but without all associated symptoms and without the long clinical course of dopamine resistance is a common presentation of acute motor deterioration in PD patients(Zheng et al. 2012).

**Table 1:**
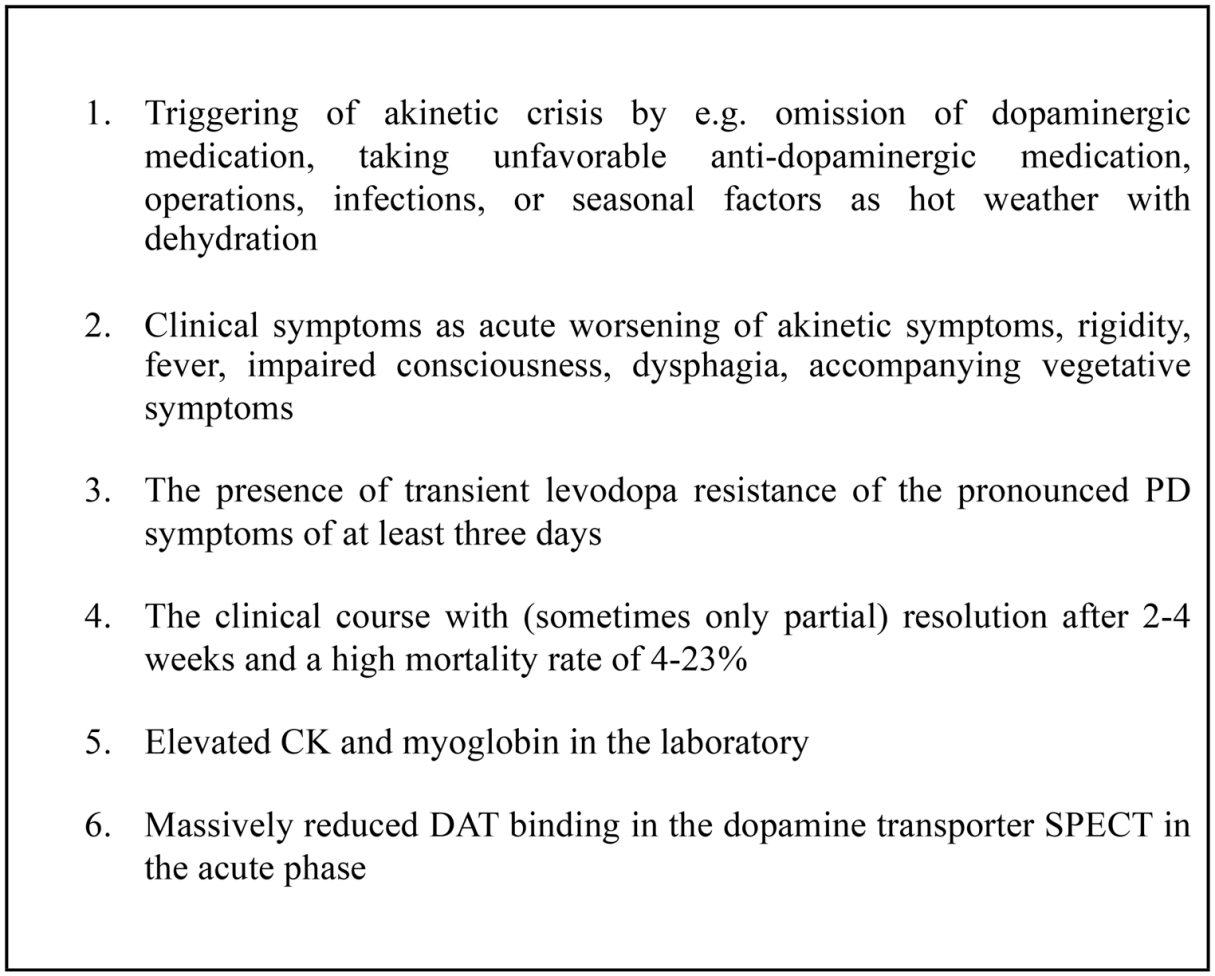
Clinical characteristics of akinectic crisis in PD

### Risk factors of akinetic crisis and subacute akinetic states

Akinetic crises were found to be more frequent in patients with advanced disease (Hoehn & Yahr > 3), hallucinations, dementia and on-off fluctuations (Takubo et al. 2003). Furthermore, genetic PD syndromes appear to have a higher risk of recurrent akinetic crises (2.12% patients/year), especially patients with genetic mutations associated with mitochondrial dysfunction such as POLG or PINK1 mutations (Bonanni et al. 2014). A recent review article based on 56 events between 1981 and 2022 described a balanced gender distribution (24 women/56 patients) as well as a variable age range (43–79 years)(Wang et al. 2022).

Trigger factors described in case reports or observational cohorts include premenstrual periods, diabetic derailment with coma, hyponatremia (Wang et al. 2022), trauma due to falls and bone fractures, postoperative complications after surgery (Onofrj and Thomas 2005) or acute bleeding anemia (Tackenberg et al. 2006), but also hot weather (Douglas and Morris 2006) and dehydration (Takubo et al. 2003). A frequent trigger factor for an akinetic crisis is the change or discontinuation of dopaminergic medication, or gastrointestinal infections or ileus, which prevent the absorption of dopaminergic medication (Onofrj and Thomas 2005; Pfeiffer and Sucha 1989). Other triggering factors include the abrupt discontinuation of amantadine (Dos Santos et al. 2021). The intake of antidopaminergic drugs such as risperidone, amisulprid or antiemetics (Bonanni et al. 2014) beside the use of antipyretics (JiYi et al. 2022) or lithium (Koehler and Mirandolle 1988) can also trigger akinetic crises. Of note, some risk factors do not seem to be fully established and variable in the rare descriptions of akinetic crises.

Probably the most frequent trigger factor for the akinetic crisis represent respiratory, urological or other systemic infections. These risk and trigger factors might exacerbate not only the akinetic crisis in its full presentation, but all the different acute-subacute akinetic states within the spectrum.

### Instrumental diagnostics of akinetic states and akinetic crisis

Additional, instrumental diagnostics should be performed to exclude differential diagnoses and screen for potential complications. In the *laboratory*, elevated creatine kinase (CK) and elevated myoglobin in serum (80%) and urine (Takubo et al. 2003) were found in 80–100% of patients with akinetic crisis. These parameters should be monitored to prevent acute renal failure due to rhabdomyolysis. Furthermore, elevated urea, liver enzymes and LDH, elevated leukocytes and CRP have also been described without the presence of a simultaneous infection (Kuno et al. 1997). Other symptomatic causes should be excluded by laboratory chemistry e.g. thyrotoxic crisis or in specific patient groups with clinical red flags autoimmune encephalitis.

*Electrophysiologically*, a general slowing was found in the EEG in less than half of the PD patients in the akinetic crisis (Takubo et al. 2003). An increase in cerebral excitability in the sense of a non-convulsive status as the cause of the disturbance of consciousness should be excluded as a competing cause.

*Imaging* as cranial computer tomography, or even better, cranial magnetic resonance imaging should be performed to exclude differential diagnoses of unconsciousness.

### The pathophysiological clue of dopamine resistance in acute akinetic states

Disease severity and duration seem to be an important risk factor for the akinetic crisis, since several studies revealed higher risk of akinetic crises in PD patients with longer disease duration or higher Hoehn and Yahr scores (Zheng et al. 2012) (Takubo et al. 2003). Still, the slow, irreversible deterioration of motor and non-motor symptoms over years due to the progressive loss of dopaminergic neurons needs to be differentiated from that subacute, mostly reversible worsening of their condition due to functional changes of dopaminergic metabolism during the akinetic crisis.

Besides, the diagnostic differentiation of the akinetic crisis from “benign”, pronounced, hypokinetic motor fluctuations, which are frequently observed in advanced Parkinson’s patients, is particularly relevant. The main distinguishing feature is the good responsiveness of akinesia to resumed dopaminergic medication in the event of “benign” motor fluctuations. The hypokinetic fluctuation can be effectively terminated by the application of adequate dopaminergic medication, whereas the akinetic crisis in the acute phase cannot be terminated immediately. This transient lack of dopaminergic responsiveness in the akinetic crisis, which can last for 1–2 weeks, has been clinically proven by observations in case series (Kuno et al. 1997) and in a larger Italian cohort (Onofrj and Thomas 2005). This difference needs to be stressed out, since it could be argued, that PD patients at an advanced stage with long disease duration have an increased risk of wearing off phenomena and the akinetic crisis might be just an extended off-fluctuation.

Consistent with this clinical observation, there were descriptions of transient DAT-SPECT (ioflupane [123I] SPECT) changes during the akinetic crisis (Martino et al. 2015; Kaasinen et al. 2014). The DAT-SPECT represents an imaging biomarker of the functional state of the presynaptic dopamine transporter, reflecting different patterns according to the clinical state from normal status (grade 5 normal scintigraphy), progressive reduction of tracer uptake (grade 4 “eagle wing”, grade 3 “mixed type”, grade 2 “egg shaped”) to completely abolished tracer uptake (grade 1 “burst striatum”) (Kahraman et al. 2012). Two small case series of serial DAT-SPECT before, during and after the akinetic crisis (Martino et al. 2015; Kaasinen et al. 2014) showed transient, almost completely abolished, striatal presynaptic dopamine transporter binding (grade 1 “burst striatum”), which partially returned to normal after the crisis. This DAT-SPECT finding was classified as so characteristic that some authors proposed using the DAT-SPECT during the akinetic crisis to establish the diagnosis (Martino et al. 2015).

**Fig. 1:**
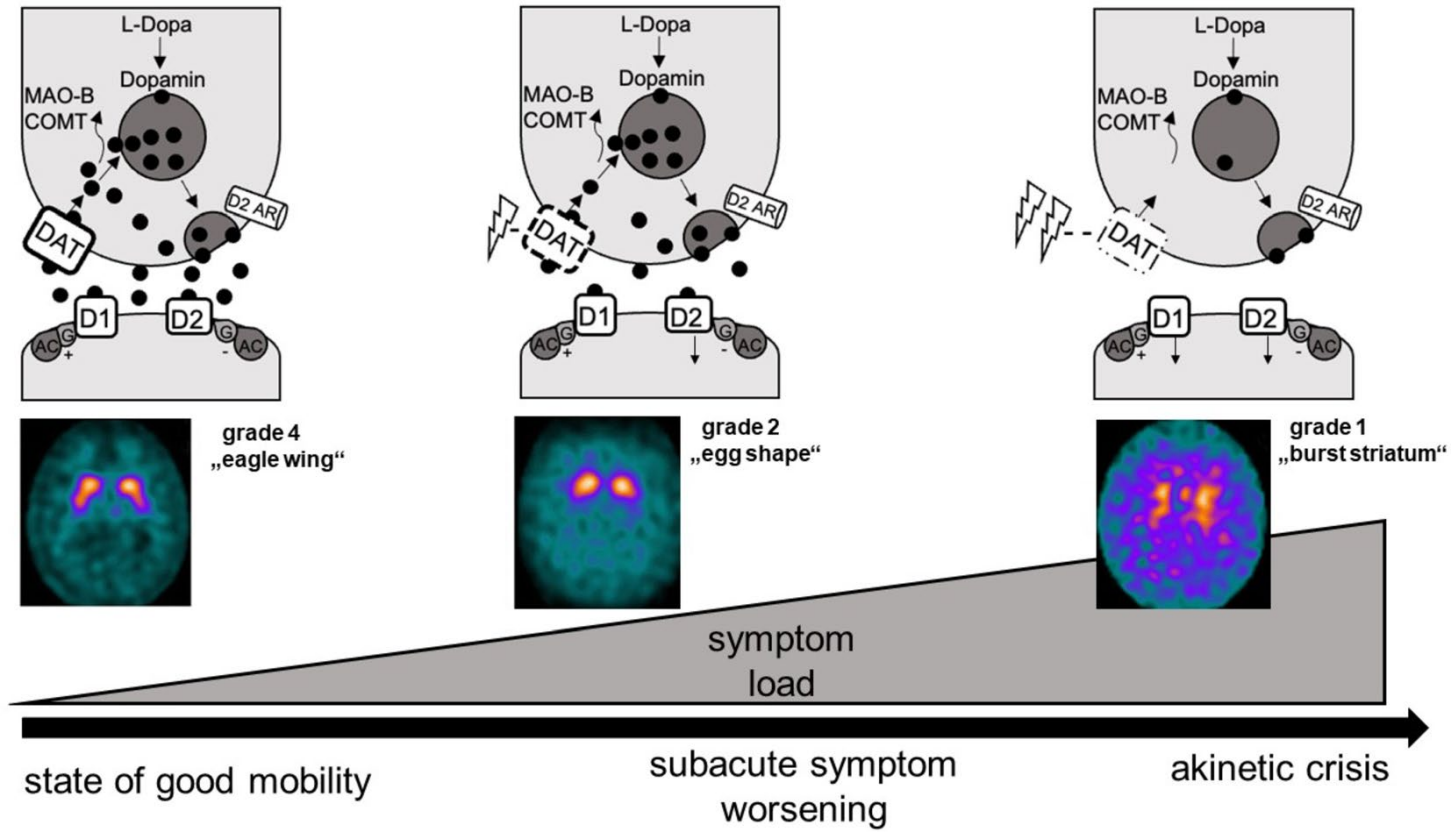
Hypotheses on progressive levodopa-resistance within the spectrum of akinetic states in PD. The simplified figure aims to synthesize potential hypotheses on clinical symptomatology in different akinetic states (lower row), functional changes of striatal synapses (upper row) and, as a linking biomarker of synaptical presynaptic dopamine transporter (DAT) availability, associated DAT-SPECT (FP-CIT SPECT) changes (middle row). Ambulatory PD patients in a state of good mobility (left column) might show slightly reduced presynaptic DAT activity for the recycling and vesical entrapment of dopamine in the presynaptic terminal of the striatum, reflected by slight changes in the DAT-SPECT (grade 4 “eagle wing”) (Kahraman et al. 2012). PD patients with subacute deterioration of motor symptoms seeking hospitalization and beginning levodopa-resistance (middle column) might reveal further reduction of DAT activity, since due to homeostatic compensatory mechanisms, decreased dopamine concentration in the intrasynaptic space induces progressive functional DAT inactivation, mirrored by further deterioration in the DAT-SPECT (grade 2 “egg shape”) (Kahraman et al. 2012). The upper end of the spectrum might be the levodopa-resistant right column akinetic crisis with nearly abolished dopamine metabolism indicated in the DAT-SPECT as “burst striatum” (grade 1) (Martino et al. 2015; Kaasinen et al. 2014)

The pathophysiological hypotheses of levodopa resistance and associated DAT-SPECT changes (Fig. 1) propose mitochondrial dysfunction at the presynaptic striatal terminal induced by neuronal stress e.g. during systemic infection. The consecutive energetic deficiency reduces the activity of the presynaptic ATP proton pump leading to dysfunction of the presynaptic dopamine transporter on the one hand and failure of dopamine release into the synaptic cleft on the other hand. The reduced synaptic dopamine level induces a vicious circle with further reduction of DAT activity in the homeostatic attempt to maintain an intrasynaptic reasonable dopamine level by compensating the reduced dopamine release into the cleft by down-regulated dopamine presynaptic reuptake (Fig. 1). Thus, the lower the intrasynaptic dopamine level, the lower the presynaptic dopamine reuptake by DAT, the larger the consecutive lack of presynaptic recyclable dopamine. Within this vicious circle, the extent of DAT functional inactivation reflects the degree of dopamine resistance associated with the severity of the akinetic state.

In summary, transient levodopa resistance is a key criterion for the diagnosis of an akinetic crisis (Onofrj and Thomas 2005) and the degree of levodopa resistance might determine the clinical severity within the spectrum of akinetic states (Fig. 1).

### Pathophysiological hypotheses of infection´s impact on acute akinetic states

Interestingly, some PD patient groups were identified in which there was no evidence of a previous change in Parkinson’s medication before the akinetic crisis, but constant levodopa plasma levels before and during the akinetic episode (Onofrj and Thomas 2005). In an Italian cohort with 26 patients developing acute akinesia, 65% of patients manifested at the onset of an infectious disease or after surgery (Onofrj and Thomas 2005). In another japanese cohort with 99 recorded akinetic events in PD patients, the second most common event were infectious diseases (19 events, 20%) (Takubo et al. 2003). In another retrospective cohort study of 120 PD patients, 43 exacerbations of subacute symptom worsening were observed with infection as the single most frequent underlying cause, accounting for 11 of 43 (25.6%) exacerbations (Zheng et al. 2012). Thus, in those epidemiological observations, infections were stressed to be one major risk factor for akinetic crises.

**Fig. 2:**
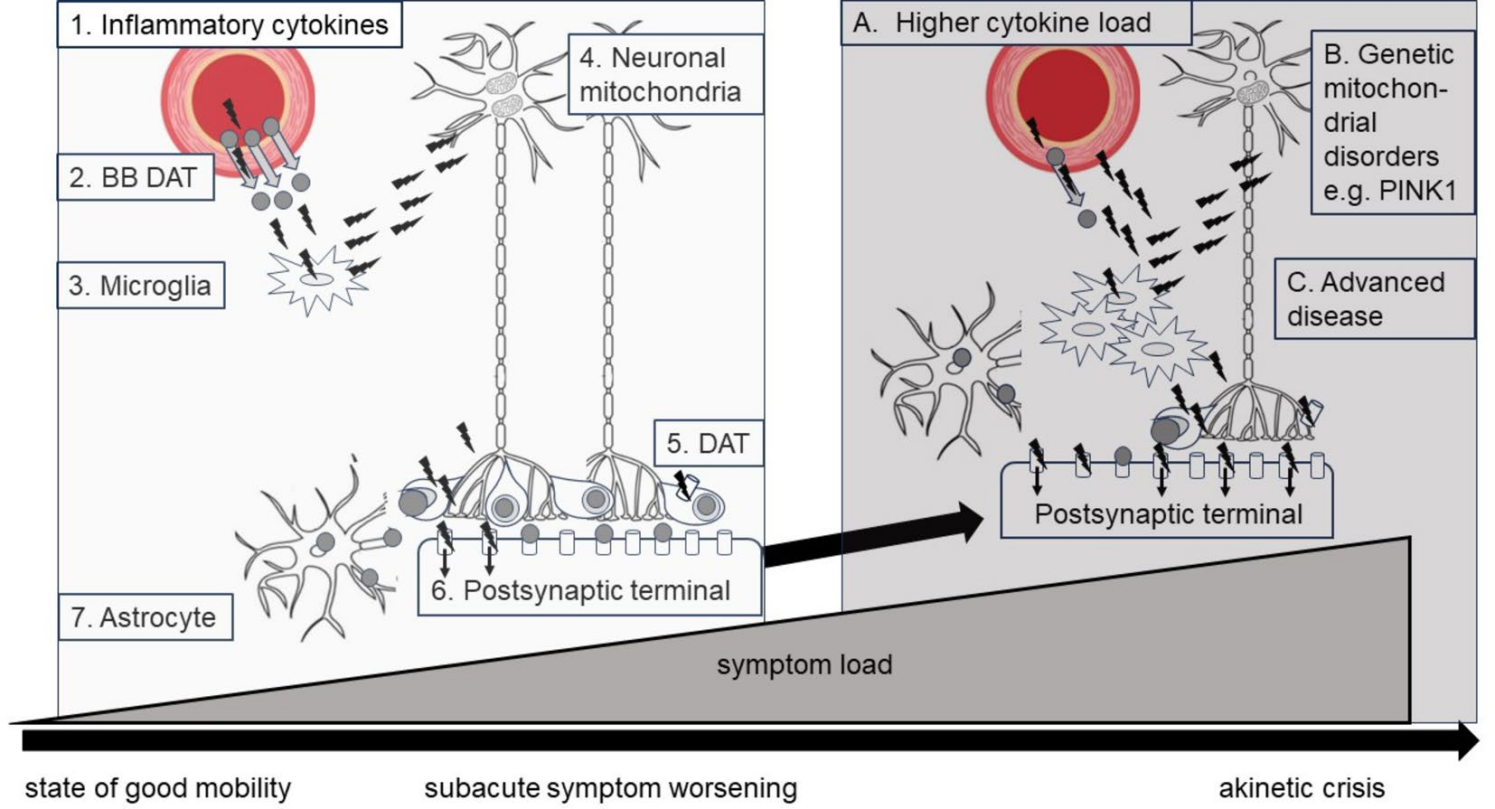
Hypotheses on the impact of proinflammatory cytokines on the evolution of levodopa-resistance in PD. The simplified figure aims to delineate the potential impact of systemic infections on progressive striatal levodopa resistance resulting in progressive akinetic states in PD patients. The invasion of proinflammatory cytokines as e.g. interleukin 6 or TNF alpha (1. black flashes) into the brain compartment might trigger a deleterious cascade leading to progressive levodopa-resistance and akinetic symptom worsening(Brugger et al. 2015). The cytokines might induce an impaired transport of levodopa through the blood-brain barrier (2.) and might activate local microglia and monocytes releasing further, multiplied loads of cytokines and maintaining the pathological inflammatory process (3.). The cytokines induce oxidative stress at the neuronal mitochondria (4.), resulting in impaired metabolism at the synapses as reduced vesicle formation and DAT activity (5.). Proinflammatory cytokines might induce internalization of postsynaptic D1 or D2 receptors (6.) and erroneous compartmentalization of dopamine into astrocytes (7.). In specific conditions those inflammatory processes might result in particularly deleterious processes leading to akinetic crisis. Higher cytokine loads in more severe septic medical conditions (**A**), predisposing genetic mitochondrial dysfunction as in e.g. PINK 1 or POLG mutation (**B**) or a more progressed disease stage with less neurons available for compensatory processes (**C**) might lead to the upper end of the spectrum of akinetic states, the akinetic crisis

A recent review described the potential chain of processes triggering levodopa resistance and akinetic crisis during infections (Brugger et al. 2015) (Fig. 2). During systemic infections, proinflammatory cytokines invade the different body compartments blood, intestinal tract and brain. They interfere with the levodopa intestinal transport to blood and alter the transport through the blood-brain barrier. Within the brain, the cytokines activate microglia and monocytes resulting in further release of additional proinflammatory cytokines and free radicals. Those oxidative stress factors impact mitochondrial function of dopaminergic nigral neurons resulting in impaired packing of dopamine into vesicles in the neuronal terminal, reduced DAT reuptake of dopamine and downregulation and internalization of postsynaptic dopaminergic receptors. Besides there is a redistribution of dopamine by unphysiological compartmentalization of dopamine into astrocytes instead of reallocation into neurons (Fig. 2). Within this pathophysiological chain of processes, there are vulnerable chain links, which might push the clinical severity of the akinetic state to the upper end of the spectrum, the akinetic crisis. The higher the load of proinflammatory cytokines, the more the inflammatory chain of processes is driven, the worse might be the clinical presentation (Fig. 2A.). Predisposing genetic risk factors as hereditary mitochondrial dysfunction as e.g. PINK1 mutation (Bonanni et al. 2014)might enhance neuronal vulnerability to oxidative stress by proinflammatory cytokines (Fig. 2B.). In advanced disease stages(Takubo et al. 2003; Wang et al. 2022), progressive neuronal degeneration result in decreased numbers of neurons in the striatal compartment with lower capability to compensate for neuronal dysfunction of neighborhood neurons (Fig. 2C). In summary, inflammatory processes induced by proinflammatory cytokines during systemic infections lead to progressive levodopa resistance resulting in graded clinical akinetic states with the upper end of the spectrum, the akinetic crisis.

### Current treatment guidelines of akinetic crisis

**Fig. 3:**
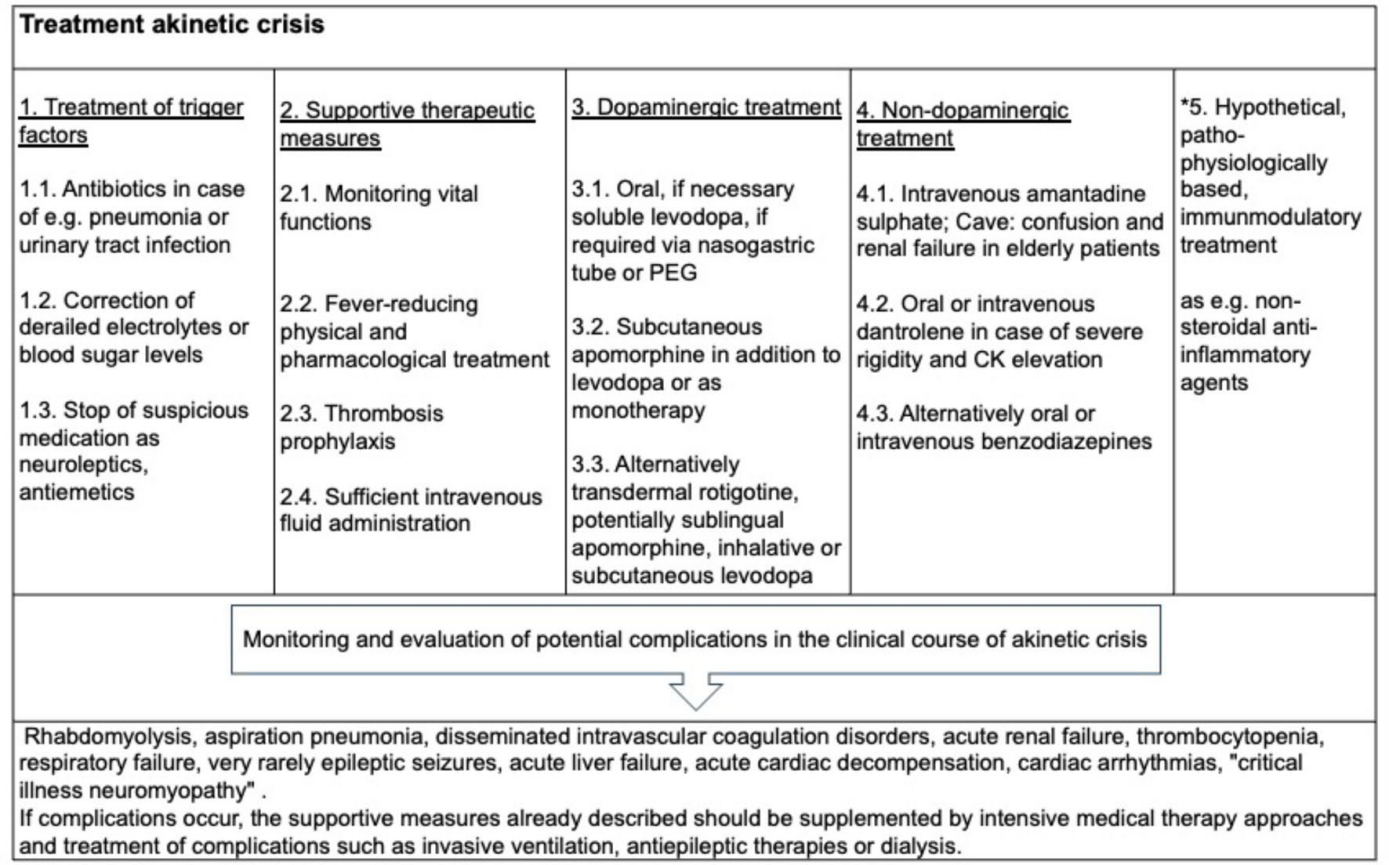
Treatment pathway of akinetic crisis in PD. The treatment should be adjusted to symptom constellation as dysphagia and be based on four pillars as treating trigger factors of akinetic crisis, supportive therapeutic measures, dopaminergic and non-dopaminergic agents. Theoretically, based on pathophysiological considerations of cytokine storms resulting in dopamine resistance, immunomodulatory approaches as e.g. NSAR could be of benefit as a fifth pillar of treatment

The treatment of PD patients during the akinetic crisis represents a particular challenge (Wang et al. 2022). Due to the pronounced dysphagia and gastrointestinal motility disorder, peroral administration of the medication is restricted. The transient levodopa resistance limits the possibilities of classic dopaminergic treatment approaches. Immobility leads to a high complication rate with intensive care treatment requirements and a high mortality rate. The therapy is based on various supportive, dopaminergic and non-dopaminergic approaches and treatment of complications (Fig. 3). It is important to start adequate therapy early, ideally under intensive care conditions, in order to improve the prognosis(Onofrj et al. 2009; Onofrj and Thomas 2005; Takubo et al. 2003).

There are several *general considerations* of supportive treatment approaches. In a first step, potential trigger factors of the akinetic crisis should be treated. Pneumonia or a urinary tract infection should be treated with antibiotics, derailed electrolytes and blood sugar levels should be corrected, triggering medication such as neuroleptics and antiemetics should be discontinued. In a second step, adequate supportive therapeutic approaches should be taken. Vital functions should be checked regularly, fever-reducing physical measures and, if necessary, medication should be used. Thrombosis prophylaxis and sufficient intravenous fluid administration should be ensured. In a third step, complications as rhabdomyolysis, acute renal failure, disseminated intravascular coagulation should be prevented, that impair the prognosis. If complications occur, the supportive therapeutic approaches should be supplemented by intensive medical therapy approaches and treatment of complications such as invasive ventilation, antiepileptic therapies or dialysis.

In terms of medication, there are *dopaminergic* and *non-dopaminergic* treatment algorithms during the akinetic crisis.

One pillar of therapy should be the resumption and adjustment of *dopaminergic medication*. In case of pronounced dysphagia, parenteral routes of administration or the use of a nasogastric tube or PEG tube are recommended to ensure effective medication delivery (Wang et al. 2022; Gordon and Frucht 2001). Transient levodopa resistance must be taken into account, which should lead to adequate dose adjustment. Levodopa is the most commonly used medication in the akinetic crisis. If nasogastric or PEG tubes are used, the interference of dopaminergic medication with gastroenteric nutrition must be taken into account, as parallel application leads to a loss of efficacy of the levodopa (Gordon and Frucht 2001). One possibility is the predominant application of tube feeding at night and dopaminergic medication during the day. Another possibility is to discontinue tube feeding 1 h before and after levodopa application. In addition, it is being discussed to choose tube feeds with a lower protein content with a protein content limit of 0.8 g/kg/day (Bonnici et al. 2010). Secondly, it should be noted that only certain levodopa preparations are compatible with tube feeding. Soluble levodopa dissolved in water can be safely administered via the tube. Thirdly, the dose of soluble levodopa must be taken into account. The patient’s medication prior to the onset of the akinetic crisis should be converted to the appropriate levodopa equivalent dose (LEDD conversion table see (Nyholm and Jost 2021) and a further dosage increase should be performed assuming transient reduction in dopa sensitivity and titration according to clinical symptoms. In specific treatment-resistant cases, a levodopa-carbidopa intestinal gel (LCIG) infusion can be considered (Newman et al. 2009). This alternative is particularly suitable if there is already an indication for PEG placement due to persistent, severe dysphagia or if LCIG therapy could also be useful after the acute crisis in case of preexisting motor fluctuations. In this case, a sufficiently large access should be selected when placing the PEG (20/9 Charrière). A further, potential therapeutical option during the akinetic crisis might be the newly approved subcutaneous foslevodopa/foscarbidopa pump (Antonini et al. 2023), however there are no data available so far.

In addition to or as an alternative to levodopa, various dopamine agonists can be used in the treatment of akinetic crisis. Apomorphine is a fast-acting dopamine agonist that can be administered via intermittent applications in the form of subcutaneous pen injections, sublingually inserted apomorphine strips, or can be used continuously in the form of pump treatment. Case reports have shown good effects of apomorphine applications (Onofrj and Thomas 2005; Auffret et al. 2019) either as monotherapy or as combination therapy with levodopa with restitution of symptoms within 24–48 h. Apomorphine doses used in the literature are highly variable and range from 0.7 to 8 mg/h as subcutaneous, continuous infusion treatment and should be adjusted according to symptoms. However, there may also be a transient lack of responsiveness to apomorphine during an acute crisis (Onofrj and Thomas 2005; Thomas et al. 2003). In a cohort of 16 Parkinson’s patients in an akinetic crisis, it was described that despite apomorphine therapy at high doses (150–200 mg/day), no motor improvement was initially observed for 2–10 days and 4 patients died despite apomorphine therapy (Thomas et al. 2003). Due to the frequent side effect of nausea, apomorphine should be combined with domperidone, which can be administered rectally in case of severe dysphagia (Galvez-Jimenez and Lang 1996). Hemolysis is a rare complication of apomorphine, in that case treatment must be discontinued. The use of transdermal application of the non-ergot dopamine agonist rotigotine in akinetic crisis has been described in individual cases (Dafotakis et al. 2009; Fiore et al. 2014). Rotigotine acts by stimulating D3 > D2 > D1 receptors. It is applied as a patch over 24 h. In the case reports, rotigotine was started with a 2 mg/24 h patch, which was quickly increased to 6 mg/24 h within 4 days (Dafotakis et al. 2009; Fiore et al. 2014) with good tolerability and efficacy with a reduction in symptoms of around 50% after 2 weeks.

Another therapeutic approach is the use of *non-dopaminergic substances*. Amantadine is particularly important as an intravenous application of amantadine sulphate in the treatment of akinetic crisis (Greulich and Fenger 1995; Danielczyk 1995; Kornhuber et al. 1993; Lange et al. 1997). The mechanism of action is predominantly antiglutamatergic via NMDA receptors, anticholinergic and weakly dopaminergic. Amantadine appears to be particularly advantageous in the levodopa-resistant phase of the akinetic crisis due to its NMDA-antagonistic effect under the pathophysiological assumption of increased excitatory glutamatergic activity (Kornhuber et al. 1993). Amantadine doses of 200 mg to maximal 600 mg can be used. Side effects might be delirium, confusion and renal failure. Intravenous administration of dantrolene has been used for muscle relaxation in severe rigidity in some patients during akinetic crisis (Kipps et al. 2005; Takubo et al. 2003). Dantrolene reduces muscle tone by blocking the release of calcium from the sarcoplasmic reticulum. Symptom-oriented dosages of 1 mg/kg to a maximum of 10 mg/kg were used. Side effects described include skin necrosis in extravasations (Kipps et al. 2005). Alternatively, the use of benzodiazepines such as diazepam to reduce muscle tone can be considered.

### Outlook

With optimized, early treatment, the prognosis of the akinetic crisis can be significantly improved. In a Japanese cohort, complete remission was achieved in 68.7% of Parkinson’s patients, but 31.3% of patients showed a permanent, residual deterioration and a mortality rate of 4% (Takubo et al. 2003). In other cohorts, a mortality rate of 23% in genetically predisposed patients (Bonanni et al. 2014) or 21.4% (Wang et al. 2022) was described. In view of the hypothesis of a spectrum of akinetic states with the akinetic crisis as the “upper end”, one would hypothesize to start adequate treatment in subacute motor deterioration as early as possible to prevent the right-shift to the upper end within the akinetic spectrum that is probably associated with progressive neuronal levodopa resistance. In view of new hypotheses of graded, transient neuronal levodopa- resistance during akinetic states due to proinflammatory cytokines during infections (Brugger et al. 2015), the hypothesis needs to be considered to administer non-steroidal anti-inflammatory drugs to reduce the cytokine storm and prevent inflammatory-related levodopa resistance leading to further motor deterioration within the spectrum of akinetic states. However, to date there are no data available to prove the efficacy of this hypothetical, therapeutical approach.
